# Novel Insights Into *MALAT1* Function as a MicroRNA Sponge in NSCLC

**DOI:** 10.3389/fonc.2021.758653

**Published:** 2021-10-27

**Authors:** Qinfeng Zhou, Lianfang Liu, Jing Zhou, Yuanyuan Chen, Dacheng Xie, Yinan Yao, Dawei Cui

**Affiliations:** ^1^ Department of Laboratory Medicine, Zhangjiagang TCM Hospital Affiliated to Nanjing University of Chinese Medicine, Zhangjiagang, China; ^2^ Department of Oncology, Zhangjiagang TCM Hospital Affiliated to Nanjing University of Chinese Medicine, Zhangjiagang, China; ^3^ Department of Medical Oncology, Shanghai Pulmonary Hospital & Thoracic Cancer Institute, Tongji University School of Medicine, Shanghai, China; ^4^ Department of Respiratory Medicine, The First Affiliated Hospital, Zhejiang University School of Medicine, Hangzhou, China; ^5^ Department of Blood Transfusion, The First Affiliated Hospital, Zhejiang University School of Medicine, Hangzhou, China

**Keywords:** long non-coding RNA, metastasis-associated lung adenocarcinoma transcript-1, non-small cell lung cancer, metastasis, invasion, microRNA

## Abstract

The long non-coding RNA metastasis-associated lung adenocarcinoma transcript-1 (*MALAT1*) was initially found to be overexpressed in early non-small cell lung cancer (NSCLC). Accumulating studies have shown that *MALAT1* is overexpressed in the tissue or serum of NSCLC and plays a key role in its occurrence and development. In addition, the expression level of *MALAT1* is significantly related to the tumor size, stage, metastasis, and distant invasion of NSCLC. Therefore, *MALAT1* could be used as a biomarker for the early diagnosis, severity assessment, or prognosis evaluation of NSCLC patients. This review describes the basic properties and biological functions of *MALAT1*, focuses on the specific molecular mechanism of *MALAT1* as a microRNA sponge in the occurrence and development of NSCLC in recent years, and emphasizes the application and potential prospect of *MALAT1* in molecular biological markers and targeted therapy of NSCLC.

## Introduction

Non-small cell lung cancer (NSCLC) is one of the leading causes of cancer-related deaths worldwide (1). Although great advances have been made in surgery, chemotherapy, and immunotherapy, the 5-year survival rate of patients with NSCLC is still only about 15% due to the high rate of distant metastasis and recurrence (2, 3). Therefore, the invasion and the metastasis of cancer cells are serious challenges in the treatment of NSCLC. In-depth understanding of the potential mechanisms of the occurrence and development of NSCLC is of great significance in order to improve the effect of clinical treatment.

Long non-coding RNA (lncRNA) is a transcript consisting of more than 200 nucleotides in length (4). It is well known that lncRNA can regulate the expressions of many genes and participate in the development of tumors (5). Metastasis-associated lung adenocarcinoma transcript-1 (*MALAT1*) was initially found to be overexpressed in early NSCLC, which is a type of non-coding ribonucleic acid (6). Although there have been many studies on *MALAT1* in the past, the specific molecular mechanism of *MALAT1* regulation of NSCLC is still not very clear (7). In the past decade, more and more studies have found that *MALAT1* can regulate its downstream target molecules by directly binding to microRNA (miRNA), thus playing an important role in the cell proliferation, metastasis, invasion, and treatment of drug resistance in NSCLC (8–11). In this review, we first briefly introduce the basic properties and biological functions of *MALAT1*, focus on the molecular mechanism of *MALAT1* as an miRNA sponge in the occurrence and the development of NSCLC, and highlight the application and potential prospect of *MALAT1* in molecular biological markers and targeted therapy in NSCLC.

## Discovery of LncRNA *MALAT1*

*MALAT1* is also termed nuclear enriched abundant transcript 2 (*NEAT2*) (12). The structure and biogenesis of its genes are located in human chromosome 11q13 and mouse chromosome 19qA (13, 14). The *MALAT1* transcript is about 7 kb in humans and 6.7 kb in mice (12, 15). Previously, *MALAT1* was named because of its clinical significance in predicting the metastasis and survival of early NSCLC, but a subsequent study showed that *MALAT1* is widely expressed in normal tissues and is extremely abundant and widely conserved in 33 species of mammals (6, 16), which indicates that *MALAT1* may have potentially important biological functions (17).

**Figure 1:**
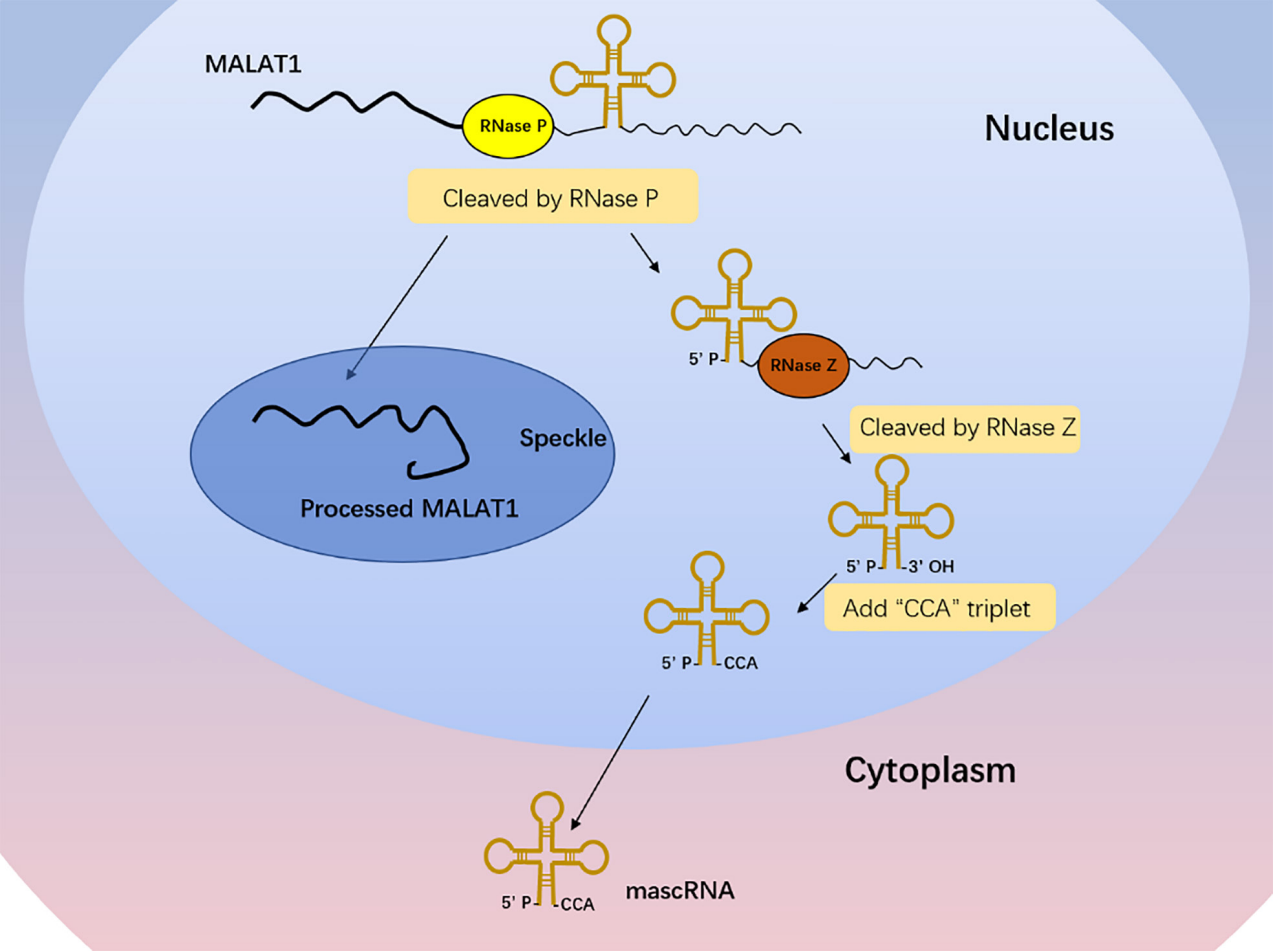
*MALAT1* biogenesis. The primary transcript of *MALAT1* forms a mature transcript of 7 kb and a small transcript fragment at the 3′ end with the cleavage of RNase P. The mature transcript is mainly located in nuclear speckles, and its 3′ end is highly conserved and forms a unique triple-helix structure that can protect it from the damage of 3′–5′ exonucleases, which is beneficial to the stability of *MALAT1*. The small transcript fragment is bound by RNase Z and further cleaved and modified by the CCA-adding enzyme to produce a 61-nt-long lncRNA called *MALAT1*-associated small cytoplasmic RNA (mascRNA), then folds into the tRNA cloverleaf structure and is exported to the cytoplasm.

Different from the typical mechanism of cleavage and polyadenylation, the *MALAT1* 3′ end lacks the structure of poly(A) tail (18). With the cleavage of ribonuclease (RNase P), the primary transcript of *MALAT1* forms a mature transcript of 7 kb and a small transcript fragment at the 3′ end (**Figure 1**) (18). The mature transcript is mainly located in nuclear bodies known as nuclear speckles, which are subnuclear structures enriched with RNA processing factors and poly(A)^+^ RNAs and involved in posttranscriptional regulation of gene expression (19, 20). Its 3′ end is highly conserved and forms a unique triple-helix structure that can protect it from the damage of 3′–5′ exonucleases, which is beneficial to the stability of *MALAT1* (21, 22). The small transcript fragment is bound by ribonuclease Z (RNase Z) and further cleaved and modified by the CCA-adding enzyme to produce a 61-nt-long lncRNA called *MALAT1*-associated small cytoplasmic RNA (mascRNA), then folds into the transfer RNA (tRNA) cloverleaf structure and is exported to the cytoplasm (**Figure 1**) (18). *MALAT1* located in nuclear speckles can regulate other physiological and pathological processes such as embryonic development, tumor progression, cardiovascular remodeling, and tissue inflammation mainly by affecting gene transcription, interfering with messenger RNA (mRNA) cleavage, regulating epigenetic changes, or acting as a competitive endogenous RNA (23–28). There are few reports on the role of mascRNA, which may participate in cardiovascular innate immunity by affecting fas ligand (FASLG), tumor necrosis factor-α (TNF-α), interleukin-6 (IL-6), etc. (29) It may also be part of the molecular mechanism of function in cancer to regulate the glutaminyl-tRNA synthetase (QARS) protein levels and promote global protein translation and cell proliferation (30).

## The Properties and Biological Functions of *MALAT1*

**Figure 2:**
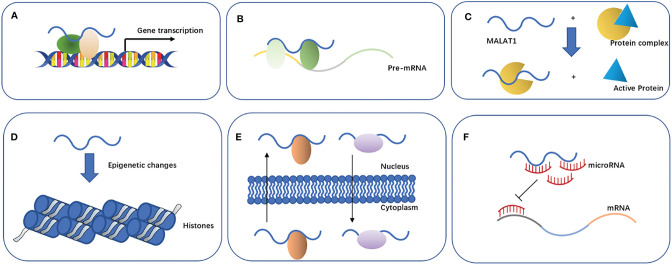
Properties and biological functions of MALAT1. **(A)** Affects gene transcription. **(B)** Affects the alternative splicing of pre-mRNAs. **(C)** Regulates protein activity. **(D)** Mediates epigenetic changes. **(E)** Promotes nuclear and cytoplasmic translocation of cellular proteins. **(F)** Acts as an endogenous miRNA sponge.

Previous studies have found that *MALAT1* can participate in the regulation of biological function through the following main mechanisms (**Figure 2**): 1) *affecting the gene transcription*. *MALAT1* can recruit Sp1, a transcription factor, in multiple myeloma. Sp1 can activate and promote the secretion of growth factor TGF-β by binding to the prompter of latent transforming growth factor beta binding protein 3 (*LTBP3*) (31). *MALAT1* can promote the transcription of telomeric repeat-binding factor 2 (*TRF2*) by recruiting RNApol II, P300, and CRUPT to bind to the promoter region of *TRF2*, which promotes the growth of liver cancer stem cells (32). 2) *Affecting the alternative splicing of pre-mRNAs*. *MALAT1* is identified as a nuclear-retained regulatory RNA that can interact with the serine- and arginine-rich (SR) protein splicing factors such as SRSF1, SRSF2, and SRSF3, affect the distribution of splicing factors in nuclear speckle domains, and regulate alternative splicing of pre-mRNAs (33). Additionally, *MALAT1* can promote ovarian cancer progression by regulating the splicing factor RBFOX2-mediated alternative splicing (34). Furthermore, *MALAT1* can induct the oncogenic splicing factor SRSF1 and modulate the alterative splicing of SK61 in hepatocellular carcinoma (35). 3) *Regulating protein activity*. *MALAT1* can competitively bind to *SFPQ* leading to *PTBP2* release from the *SFPQ*/*PTBP2* complex, which enhances the function of *PTBP2* in promoting tumor cell proliferation and migration (36). 4) *Mediating epigenetic changes*. Malat1 can cause the trimethylation of histone 3 lysine 9 (H3K9me3) by recruiting the suppressor of variegation 3–9 homolog 1 (Suv39h1) to MyoD-binding loci. This trimethylation suppresses the transcriptional activity of MyoD, which represses myoblast differentiation (37). In addition, the overexpression of *MALAT1* could increase the expression of acetyl-H4 histone in the IQ motif-containing GTPase-activating protein 1 (IQGAP1) promoter, which may promote the proliferation and invasion of thyroid cancer cells (38). 5) *Promoting the nuclear and cytoplasmic translocation of cellular proteins*. *MALAT1* retains the serine/arginine-rich proteins SF2/ASF from the cytoplasm to the nucleus, thus promoting the development of gastric cancer cells (39). *MALAT1* can bind to an abundant nuclear factor heterogeneous nuclear ribonucleoprotein C (hnRNPC) protein, which could transfer from the nucleus to the cytoplasm during cell division, to assist its translocation (40). (6) *Acting as an endogenous miRNA sponge*. MiRNAs play an important role in cell proliferation, differentiation, apoptosis, and development. Recent evidence suggests that other RNAs such as lncRNA can also compete with mRNAs by sponging miRNAs (41). Among these lncRNAs, *MALAT1* is one of the most studied RNAs involved in various molecular processes such as endogenous miRNA sponging (42). Here, we will focus on the potential function of *MALAT1* as a miRNA sponge in NSCLC (**Table 1**).

**Table 1:** Mechanism and roles of the metastasis-associated lung adenocarcinoma transcript-1 (*MALAT1*) in non-small cell lung cancer (NSCLC) progression.

miRNA	Target genes of miRNA	Downstream pathways	Biological functions	Reference
miR-1914-3p	*YAP*	METTL3/*MALAT1*/miR-1914-3p/YAP	Promote drug resistance and tumor metastasis	(11)
miR-197-3p	p120-ctn	*MALAT1*/miR-197-3p/p120-ctn	Promote proliferation, viability, and EMT of NSCLC and depress chemosensitivity and apoptosis	(43)
miR-142-3p	β-catenin	miR-142-3p/*MALAT1*/β-catenin	Promote proliferation, invasion, and tumor formation and inhibit apoptosis	(44)
miR-206	–	*MALAT1*/miR-206/Akt/mTOR signaling	Promote NSCLC cell migration and invasion	(45)
miR-124	*STAT3*	*MALAT1*/miR-124/STAT3	Promote the progression of NSCLC	(46)
miR-200a-3p	*PD-L1*	*MALAT1*/miR-200a-3p/PD-L1	Promote proliferation, mobility, migration, and invasion	(10)
miR-145	*KLF4*	*MALAT1*-miR-145-KLF4	Induce cisplatin resistance	(47)
miR-185-5p	*MDM4*	*MALAT1*/miR-185-5p/MDM4	Promote proliferation, migration, and invasion and impede apoptosis	(48)
miR-515-5p	EEF2	*MALAT1*/miR-515-5p/EEF2	Promote proliferation and invasion and reduce apoptosis	(49)
miR-146a/miR-216	*BRCA1*	*MALAT1*/miR-146a/miR-216/BRCA1	Participate in the DNA repair process of NSCLC cells and attenuate cisplatin sensitivity	(50)
miR-145-5p	*NEDD9*	ERβ/*MALAT1*/miR-145-5p/NEDD9	Promote VM and cell invasion	(51)
miR-374b-5p	*SRSF7*	*MALAT1*/miR-374b-5p/SRSF7	Promote proliferation and migration and inhibit apoptosis	(7)
miR-613	*COMMD8*	*MALAT1*/miR-613/COMMD8	Promote proliferation, colony formation, and glycolysis and attenuate apoptosis	(52)
miR-101-3p	*MALAT1*	miR-101-3p/*MALAT1*/PI3K/AKT signaling	Promote growth and metastasis of NSCLC	(53)

EMT, epithelial–mesenchymal transition; VM, vasculogenic mimicry.

## Mechanism of *MALAT1* in NSCLC Progression as a MicroRNA Sponge

### miR-1914-3p

*N* ^6^-methyladenosine (m6A) mRNA methylation initiated by methyltransferase-like 3 (METTL3) promotes the translation of YAP mRNA by recruiting *YTHDF1*/*3* and eIF3b into the translation initiation complex, so the expression of METTL3 is positively correlated with the level of YAP protein (54). On the other hand, METTL3 improved the m6A modification level of the lncRNA *MALAT1* and increased its stability. *MALAT1* sponging miR-1914-3p weakens the ability of miR-1914-3p to target and inhibit YAP, thus increasing the expression of YAP in NSCLC (54). The increased expression and activity of YAP lead to cisplatin (DDP) resistance and metastasis of NSCLC (54). Therefore, the increased activity of the METTL3/*MALAT1*/miR-1914-3p/YAP axis promotes the metastasis and drug resistance of NSCLC.

### miR-197-3p

The high expressions of *MALAT1* and miR-197-3p were closely related to the survival and growth of NSCLC (43). Luciferase activity assay showed that *MALAT1* was complementary to miR-197-3p at certain sites. P120 catenin (p120-ctn) regulates the proliferation of cancer cells by regulating cell adhesion and the cell cycle (55). Yang et al. found that p120-ctn was confirmed to be a targeted downstream molecule of *MALAT1* and miR-197-3p (43). Reducing the expression of p120-ctn can repress the epithelial–mesenchymal transition (EMT) and the survival and proliferation ability of NSCLC, while it enhances the apoptosis rate of cancer cells. Moreover, p120-ctn can mediate the role of *MALAT1* and miR-197-3p in promoting the progression and chemotherapy resistance of NSCLC cells (43). The results of *in vivo* experiments using NSCLC mouse models showed that a low expression of *MALAT1*, miR-197-3p, or p120-ctn can decrease the tumor volume and weight compared with the control group (43). Consequently, the *MALAT1*/miR-197-3p/p120-ctn axis may play a potential role in the regulation of NSCLC, which will provide a direction for improving the prognosis of NSCLC patients after chemotherapy.

### miR-142-3p

The expression of miR-142-3p decreased, while β-catenin and *MALAT1* increased in NSCLC tissues. RT-PCR and luciferase reporter assays showed that miR-142-3p negatively inhibited the level of *MALAT1* by directly binding to the 3′-UTR of *MALAT1* mRNA (44). On the one hand, upregulation of miR-142-3p mimic transfection can significantly reduce the proliferation and migration of NSCLC H1299 cells while inducing G0/G1 phase arrest and reducing that of the S phase; on the other hand, the overexpression of miR-142-3p can downregulate the expression of β-catenin in H1299 cells (44). *In vivo* experiments showed that the upregulation of miR-142-3p and the downregulation of β-catenin or *MALAT1* could significantly reduce the tumorigenicity of NSCLC cells (44). To sum up, miR-142-3p can play a tumor-suppressing role in the progression of NSCLC by inhibiting the *MALAT1*/β-catenin signaling pathway.

### miR-206

Tang et al. detected the expression of *MALAT1* in tumor tissues and adjacent normal tissues in 36 cases of NSCLC using real-time quantitative PCR (qRT-PCR) and found that the expression of *MALAT1* was significantly upregulated in NSCLC tissues (45). In addition, *MALAT1* promoted EMT, cell migration, and invasion by activating the Akt/mTOR signals in A549 and H1299 cells. MiR-206 is the direct downstream target of *MALAT1* in NSCLC, and there was a negative correlation between the expressions of *MALAT1* and miR-206 in NSCLC (45). *MALAT1* promoted cell migration and invasion in NSCLC cells by sponging miR-206. In addition, miR-206 could also inhibit the activation of the Akt/mTOR signal mediated by *MALAT1* in A549 and H1299 cells (45). Taken together, *MALAT1* can promote the migration and invasion of NSCLC by targeting miR-206 and activating the Akt/mTOR signaling pathway, which provides a molecular basis for the metastasis of *MALAT1* in NSCLC.

### miR-124

It was found that the level of miR-124 in A549, H23, H522, H1299, and H460 NSCLC cells was significantly downregulated (46). Luciferase reporter assays showed that miR-124 is the direct target of *MALAT1*, and there was a potential negative correlation between miR-124 and *MALAT1*. shMALAT1 can suppress the proliferation, colony formation, and apoptosis of NSCLC cells, while miR-124 inhibitors can reverse this effect. In addition, it was also found that *STAT3* is a new mRNA target of miR-124 (46). The downregulation of *MALAT1* can inhibit the development of NSCLC by enhancing the expression of miR-124 and reducing the expression of *STAT3* (46). In summary, it is speculated that *MALAT1* may participate in the occurrence and development of NSCLC as an endogenous miRNA sponge through the *MALAT1*/miR-124/*STAT3* signaling axis.

### miR-200a-3p

The targeting relationship between *MALAT1* and miR-200a-3p and programmed death-ligand 1 (PD-L1) was further verified by qRT-PCR and dual-luciferase reporter gene detection (10). The researchers found that *MALAT1* sponged miR-200a-3p, and PD-L1 was identified as the target of miR-200a-3p and indirectly regulated by *MALAT1*. Moreover, the level of *MALAT1* was negatively correlated with the expression of miR-200a-3p in NSCLC, but positively correlated with the expression of PD-L1 (10). Furthermore, *MALAT1* promoted the proliferation, migration, and invasion of NSCLC cells through sponging miR-200a-3p (10). Overall, *MALAT1* promotes the progress of NSCLC by regulating the miR-200a-3p/PD-L1 axis, which is of positive significance to the selection of new targeted drugs and the enrichment of therapeutic methods in the future.

### miR-145

Kruppel-like factor 4 (KLF4) has been shown to be associated with DDP resistance in some cancers (56, 57). KLF4 is negatively regulated by miR-145 and positively regulated by *MALAT1* at the mRNA and protein levels in NSCLC A549 cells. Luciferase reporter assay, qRT-RCR, and Western blotting confirmed that *MALAT1* indirectly regulated KLF4 by directly sponging miR-145, suggesting that *MALAT1* may be involved in DDP resistance by regulating the level of KLF4 (47). In addition, *MALAT1* knockout reversed the resistance of A549rCDDP cells to DDP. Collectively, the *MALAT1*/miR-145/KLF4 axis is an important inducer of DDP resistance in NSCLC (47). Therefore, *MALAT1* may serve as a promising predictor and therapeutic target of DDP in patients with NSCLC.

### miR-185-5p

Wang et al. found that the expressions of *MALAT1* and *MDM4* were significantly high in 30 cases of NSCLC, and *MALAT1* could positively regulate the expression of *MDM4* in NSCLCs cells (48). The deletion of *MALAT1* and *MDM4* could significantly decrease the proliferation and metastasis of NSCLC cells and promote apoptosis. In addition, the binding sites of miR-185-5p and *MALAT1* or *MDM4* were predicted using a database, and their relationship was further confirmed by dual-luciferase report assays. The results showed that miR-185-5p can be a target of *MALAT1* and could also directly regulate *MDM4*, and its overexpression can obviously suppress NSCLC cells (48). It was further confirmed that *MALAT1* can promote the proliferation, migration, invasion, and apoptosis of NSCLC cells by regulating the expression of *MDM4* mediated by miR-185-5p (48). These results may provide not only a new regulatory mechanism but also a new potential therapeutic target for the treatment of NSCLC.

### miR-146a/miR-216

It has been reported that *MALAT1* is involved in the repair pathway of DNA double-strand breaks, and targeting *MALAT1* can induce apoptosis in myeloma cells (58). BRCA1 is a multifunctional protein that plays a key role in the homologous recombination DNA repair pathway (59). Through the *MALAT1* pull-down assay, the researchers found that miR-146a and miR-216 directly interact with *MALAT1* in A549 and H1299 cells and that they can specifically inhibit the expression of BRCA1 (50). By inhibiting *MALAT1*, miR-146a and miR-216 can be released to further inhibit the expression of BRCA1 and induce DNA damage. Therefore, *MALAT1* can participate in the DNA repair process of NSCLC cells by regulating the miR-146a/miR-216/BRCA1 pathway. In addition, targeting *MALAT1* can also increase the sensitivity of NSCLC cells to DDP (50). In summary, *MALAT1* may become a new target for the treatment of NSCLC.

### miR-145-5p

Estrogen receptor beta (ERβ) may affect the progression of NSCLC (51). Yu et al. found that ERβ can increase the expression of *MALAT1* by directly binding to the estrogen response elements (EREs) located on the *MALAT1* promoter, thus inhibiting miR-145-5p. Because miR-145-5p directly targets the 3′-UTR of the neural precursor cell expressed, developmentally downregulated 9 (*NEDD9*) mRNA, increasing the expression of *MALAT1* can indirectly upregulate the protein expression of *NEDD9*. Further experiments showed that ERβ could promote the vasculogenic mimicry (VM) formation and cell invasion of NSCLC by the ERβ/*MALAT1*/miR-145-5p/*NEDD9* signaling pathway (51). This may help in providing new strategies to better inhibit the metastasis of NSCLC in the future.

### miR-374b-5p

The expressions of *MALAT1* and serine/arginine-rich splicing factor 7 (*SRSF7*) were upregulated and the expression of miR-374b-5p was downregulated in NSCLC (7). The expression of *MALAT1* was negatively correlated with the expression of miR374b-5p and positively correlated with the expression of *SRSF7*. MiR-374b-5p is the target of *MALAT1*. Knockout of *MALAT1* and miR-374b-5p overexpression can inhibit the proliferation, migration, and invasion of NSCLC cells and induce apoptosis. *In vivo* experiments showed that the overexpression of *MALAT1* promoted the tumor growth of NSCLC (7). *SRSF7* is the downstream target molecule of miR-374b-5p. The overexpression of *SRSF7* reverses the effects of *MALAT1* gene knockout on the proliferation, apoptosis, migration, and invasion of NSCLC cells (7). Therefore, it was concluded that *MALAT1* participates in the progress of NSCLC through the *MALAT1*/miR-374b-5p/*SRSF7* axis. This study may provide a theoretical basis for the diagnosis and treatment of NSCLC.

### miR-613

The expressions of *MALAT1* and *COMMD8* were abnormally increased in NSCLC tissues and cells (52). We found that miR-613 is the target of *MALAT1* and that it can bind to the 3′-UTR of *COMMD8*. *MALAT1* upregulated the level of *COMMD8* by competitively targeting miR-613, thus playing a carcinogenic role in NSCLC (52). *MALAT1* or *COMMD8* gene knockout inhibited cell proliferation, clone formation, and glycolysis, but promoted cell apoptosis. *In vivo* experiments have shown that *MALAT1* gene knockout reduced the tumor growth. In addition, researchers also found that extracellular *MALAT1* was released by packaging into exosomes (52). These pieces of evidence provide new insights into the treatment of NSCLC, and the *MALAT1*/miR-613/*COMMD8* axis will be a promising approach for future treatment options.

### miR-101-3p

The relative expression of miR-101-3p in NSCLC cells decreased significantly, while the relative expression of *MALAT1* increased significantly (53). MiR-101-3p can significantly inactivate the PI3K/AKT pathway; inhibit the expression of Bcl-2 and MMP-9; and suppress the proliferation, migration, and invasion of NSCLC cells by directly binding to *MALAT1* (53). On the contrary, the overexpression of *MALAT1* reversed the inhibitory effect of miR-101-3p on the activation of the PI3K/AKT signaling pathway and the expressions of Bcl-2 and MMP-9 in NSCLC. These results suggest that miR-101-3p blocks the PI3K/AKT signaling pathway by targeting the inhibition of *MALAT1*, thus inhibiting the growth and metastasis of NSCLC (53). Therefore, miR-101-3p is expected to become an effective target for the prevention and treatment of NSCLC.

## Application of *MALAT1* in NSCLC

Although there are many methods for the diagnosis of NSCLC, these may not fully meet the needs of early diagnosis of the cancer. *MALAT1* is a relatively stable RNA transcript with a half-life of 9–12 h, which may be due to its triple-helix structure at the 3′-end (21, 22, 60). This characteristic of having a long half-life makes *MALAT1* easy to detect in tumor tissues and body fluids. Research has shown that *MALAT1* can be used as a biomarker for the diagnosis of many kinds of malignant tumors (61–63). Especially in NSCLC, the high expression of *MALAT1* was significantly correlated with tumor node metastasis (TNM) stage, vascular invasion, pathological differentiation, and recurrence (64). Further studies have shown that the overexpression of *MALAT1* was significantly related to the prognosis of lung squamous cell carcinoma, which is one type of NSCLC (65). Moreover, different expression levels of *MALAT1* in peripheral blood were observed between cancer patients and healthy controls (66).

Rong et al. found that the levels of *MALAT1* in serum exosomes were higher in patients with NSCLC, suggesting that exosome-derived *MALAT1* may also reflect the biological changes of NSCLC cells (49). Zhang et al. found that the expression of *MALAT1* in serum exosomes of NSCLC patients was upregulated and that the level of exosomal *MALAT1* was positively correlated with tumor stage and lymph node metastasis (67). The above data suggest that *MALAT1* in exosomes may also be used as a serum-based tumor biomarker to diagnose and predict NSCLC. Liquid biopsy provides the opportunity of detecting and monitoring cancer in various body fluids by detecting free circulating tumor cells, circulating tumor DNA fragments, circulating RNA, and exosomes (68). Its advantage lies in that it can reduce the harm of biopsy through noninvasive sampling and has important significance for the early diagnosis of cancer, but the low expression level of *MALAT1* in blood makes sensitive analysis difficult (66). Although some progress has been made in the detection of *MALAT1* in blood with traditional RT-PCR, the procedure is complicated, the amount of serum required is large, and the equipment is expensive. A recent study by Chen et al. showed that the detection of the levels of *MALAT1* in blood was more rapid, sensitive, and inexpensive when using a novel ultrasensitive screen-printed carbon electrode (SPCE)-based electrochemical biosensor that uses a Au nanocluster (NC)/multi-walled carbon nanotube (MWCNT)–NH_2_ nanostructure (69). This new methodology for the detection of *MALAT1* will increase its applicability to clinical diagnosis of NSCLC.

In addition, the expression level of *MALAT1* can also be used as a biomarker of chemosensitivity in different cancers (43, 70–72). Resistance to multiple drugs is the main cause of chemotherapy failure in patients with lung cancer (73). Studies have shown that *MALAT1* is also involved in the drug resistance of NSCLC. For example, Fang et al. found that the expression of *MALAT1* was upregulated in DDP-resistant A549 cells. *MALAT1* upregulated MRP1 and MDR1 by activating *STAT3*, thus reducing the sensitivity to DDP *in vitro* and *in vivo* (74). NSCLC patients carrying epidermal growth factor receptor (EGFR) mutations initially respond to EGFR tyrosine kinase inhibitors (EGFR-TKIs) such as gefitinib, but gradually developed acquired drug resistance (75, 76). It was found that the overexpression of *MALAT1* could eliminate not only the inhibitory effect of polyphyllin I (PPI) on the activity of gefitinib-resistant NSCLC cells but also the apoptosis induced by PPI, while *MALAT1* gene knockout could enhance the inhibition and apoptosis induced by PPI (77). These data suggest that *MALAT1* may represent a candidate biomarker and therapeutic target for chemotherapy drug resistance.

Due to the enrichment and high expression of *MALAT1* in the nucleus, its effect on traditional shRNAs or siRNAs may not be ideal and prone to off-target effects (78). The application of antisense oligonucleotides (ASOs) is a valuable method to antagonize *MALAT1*. ASOs, which are small RNA/DNA-based oligonucleotides capable of crossing cell membranes and binding to the target RNA in the nucleus and cytoplasm, are divided into two main categories: mixmeRs and gapmeRs (79, 80). Gutschner et al. found that *MALAT1* could be targeted with second-generation ASOs, thus leading to the drastic reduction of lung cancer metastasis in a pulmonary metastatic model *in vivo* (78). Moreover, the same investigators achieved functional knockout of *MALAT1* through zinc finger nuclease (ZFN)-mediated site-specific integration of RNA destabilizing elements into the human genome, which showed efficient silencing of the highly abundant *MALAT1* in human lung cancer cells (78).

## Conclusion and Prospects

As an important and highly conserved lncRNA, *MALAT1* has been widely studied, especially its role in tumorigenesis, metastasis, drug resistance, and clinical prognosis (81–83). However, the specific role of *MALAT1* in the occurrence and development of NSCLC has not been fully elucidated. Based on the basic biological properties of *MALAT1*, more and more studies have shown that it can be used as a bait for miRNA to share miRNA response elements (MREs) with mRNAs, which indirectly affects the expression of some specific downstream genes, thus promoting the proliferation, invasion, apoptosis, drug resistance, and tumor growth of NSCLC. In general, *MALAT1* is mostly known to be enriched in nuclear speckles, and we also agree that cytoplasmic P-bodies are the localizing site of the RNA-induced silencing complex (RISC) effector proteins Ago1–4 and the functional site of miRNA-mediated gene silencing (84). The vast majority of researchers used to apply bioinformatics program such as ChipBase, LncRNAdb, and StarBase to predict the interaction between *MALAT1* and miRNA in previous research on *MALAT1* as a miRNA sponge in NSCLC. Subsequently, they verified the direct interaction using luciferase reporter, RNA immunoprecipitation (RIP), and *MALAT1* pull-down assays. However, there was little focus on the sites (cytoplasm or nucleus) where these interactions occur. On the contrary, Jin et al. demonstrated that *MALAT1* and miR-1914-3p are abundant and stable in the cellular cytoplasm using RNA fluorescence *in situ* hybridization assay and confirmed that *MALAT1* directly binds miR-1914-3p using luciferase reporter assay, RIP for argonaute 2 (Ago2) in A549 cells, and RNA pull-down assay (54). Additionally, Leucci et al. showed that miR-9 targets *MALAT1* for degradation in the nucleus by directly binding to two miRNA binding sites (85). Furthermore, Wu et al. found that Ago2 was expressed both in the nucleus and cytoplasm of sw480 cells (86). Moreover, Gagnon et al. reported that 75% of the miRNAs in the cytoplasm could shuttle into the nucleus and then bind to nuclear Ago2 (87). These studies showed that the distribution of *MALAT1* or miRNA is not limited to the nucleus or cytoplasm. Hence, we wondered whether *MALAT1* or miRNA might be involved in some cases with nucleoplasmic translocation. Additionally, the locations of *MALAT1* and various miRNA interactions in NSCLC cells need to be further verified and explored.

Taken together, based on the literature, some miRNAs such as miR-142-3p and miR-101-3p can target *MALAT1* for degradation, thereby negatively inhibiting the lever of *MALAT1* in NSCLC (44, 53). On the contrary, *MALAT1* can also act as a miRNA sponge by sequestering the target miRNAs and affecting downstream gene expression, and the expression level of *MALAT1* was negatively correlated with the expressions of miRNAs in NSCLC (53). Whether miRNA is degraded or recycled remains to be investigated. It also has been reported that *MALAT1* and some miRNAs were more abundant in the Ago2 pellet than in the immunoglobulin G (IgG) pellet by conducting an RIP assay, which suggested that *MALAT1* might be a target of miRNA through an Ago2-dependent manner.

Intriguingly, there is an exosome-derived *MALAT1* in the serum of NSCLC patients, and the expression of *MALAT1* in exosomes is highly correlated with the TNM stage and lymphatic metastasis of NSCLC. However, at present, the mechanism of *MALAT1* in the exosomes of NSCLC patients remains in the preliminary research stage and needs to be further clarified. It is interesting to note that, due to the enrichment and high expression of *MALAT1* in the nucleus, the specific mechanism of *MALAT1* packing into exosomes that are rarely reported remains to be explored in the future, although it is common for lncRNA as a cargo to be loaded into exosomes. Moreover, *MALAT1* may be a key actor in the hallmark of resisting cell death as it can decrease the levels of cleaved CASP3 in NSCLCs, which leads to escaping apoptosis (77, 88). On the contrary, whether *MALAT1* detection in serum due to cell death may involve complex mechanisms needs to be further studied.

In addition, *MALAT1* knockout mice did not cause obvious phenotype in development, gene expression, and physiological function, which is not consistent with *MALAT1* being involved in the occurrence and development of NSCLC *in vitro*, so this also needs to be further explored (89). In-depth understanding of the function and regulatory mechanism of *MALAT1* in NSCLC may provide a new breakthrough for the diagnosis and targeted therapy of NSCLC in the future.

## Author Contributions

QZ wrote the manuscript and designed the figures. DC constructed the topic. LL, JZ, and YC provided scientific suggestions and participated in manuscript preparation. DX and YY provided guidance and revised this manuscript. All authors contributed to the article and approved the submitted version.

## Funding

The study was supported by grants provided by The National Natural Science Foundation of China (81871709), the Natural Science Foundation of Suzhou (KJXW2017063), Natural Science Foundation of Zhangjiagang (ZKS2022).

## Conflict of Interest

The authors declare that the research was conducted in the absence of any commercial or financial relationships that could be construed as a potential conflict of interest.

## Publisher’s Note

All claims expressed in this article are solely those of the authors and do not necessarily represent those of their affiliated organizations, or those of the publisher, the editors and the reviewers. Any product that may be evaluated in this article, or claim that may be made by its manufacturer, is not guaranteed or endorsed by the publisher.

## Abbreviations

NSCLC, non-small-cell lung cancer; LncRNA, long non-coding RNA; *MALAT1*, metastasis-associated lung adenocarcinoma transcript-1; *NEAT2*, nuclear enriched abundant transcript 2; RNase, ribonuclease; FASLG, fas ligand; TNF-α, tumor necrosis factor-α; IL-6, interleukin-6; QARS, glutaminyl-tRNA synthetase; *LTBP3*, latent transforming growth factor beta binding protein 3; *TRF2*, transcription of telomeric repeat-binding factor 2; H3K9me3, trimethylation of histone 3 lysine 9; Suv39h1, suppressor of variegation 3–9 homolog 1; IQGAP1, IQ motif-containing GTPase-activating protein 1; hnRNPC, heterogeneous nuclear ribonucleoprotein C; m6A, *N* ^6^-methyladenosine; METTL3, methyltransferase-like 3; DDP, cisplatin; p120-ctn, P120 catenin; EMT, epithelial–mesenchymal transition; qRT-PCR, real-time quantitative PCR; PD-L1, programmed death-ligand 1; KLF4, Kruppel-like factor 4; ERβ, estrogen receptor β; EREs, estrogen response elements; VM, vasculogenic mimicry; *SRSF7*, serine/arginine-rich splicing factor 7; EGFR-TKI, epidermal growth factor receptor tyrosine kinase inhibitor; PPI, polyphyllin I; ASOs, antisense oligonucleotides; ZFN, zinc finger nuclease; MRE, miRNA response element; RIP, RNA immunoprecipitation.
